# Combination of Mac-2 Binding Protein Glycosylation Isomer and Up-To-Seven Criteria as a Useful Predictor for Child-Pugh Grade Deterioration after Transarterial Chemoembolization for Hepatocellular Carcinoma

**DOI:** 10.3390/cancers11030405

**Published:** 2019-03-22

**Authors:** Yuji Eso, Atsushi Takai, Ken Takahashi, Yoshihide Ueda, Kojiro Taura, Hiroyuki Marusawa, Hiroshi Seno

**Affiliations:** 1 Department of Gastroenterology and Hepatology, Graduate School of Medicine, Kyoto University, 54 Shogoin-Kawaharacho, Sakyo-ku, Kyoto 606-8507, Japan; atsushit@kuhp.kyoto-u.ac.jp (A.T.); takaken@kuhp.kyoto-u.ac.jp (K.T.); yueda@kuhp.kyoto-u.ac.jp (Y.U.); seno@kuhp.kyoto-u.ac.jp (H.S.); 2 Department of Surgery, Graduate School of Medicine, Kyoto University, 54 Shogoin-Kawaharacho, Sakyo-ku, Kyoto 606-8507, Japan; ktaura@kuhp.kyoto-u.ac.jp; 3 Department of Gastroenterology and Hepatology, Osaka Red Cross Hospital, 5-30 Fudegasaki-cho, Tennoji-ku, Osaka 543-8555, Japan; maru@kuhp.kyoto-u.ac.jp

**Keywords:** Child-Pugh grade, hepatocellular carcinoma, Mac-2-binding protein glycosylation isomer, transarterial chemoembolization, up-to-seven criteria

## Abstract

Transarterial chemoembolization (TACE) is the recommended first-line treatment for intermediate-stage hepatocellular carcinoma (HCC). In patients who became refractory to TACE, a treatment switch to tyrosine kinase inhibitors (TKIs) needs to be considered. However, TACE could worsen liver function, thereby narrowing the time window for a switch to TKIs because TKIs are recommended for patients with Child-Pugh grade A (CP-A). We investigated the factors associated with CP grade deterioration after TACE. Among patients who underwent TACE, 125 patients with CP-A were enrolled. The cumulative CP grade deterioration rates were 20.3%, 27.1%, and 41.4% at six months, one year, and two years, respectively. Multivariate analysis revealed that factors associated with CP grade deterioration included high Mac-2 binding protein glycosylation isomer (M2BPGi) levels (>2.00 cut-off index) and beyond the up-to-seven criteria. The cumulative CP grade deterioration rates of patients with high M2BPGi and beyond the up-to-seven criteria were 50.6% and 59.2% at six months and one year, respectively, which were significantly higher than for those in any other groups. The combination of M2BPGi and up-to-seven criteria could be a surrogate marker for predicting CP grade deterioration after TACE. In patients with intermediate-stage HCC, elevated M2BPGi levels, and beyond the up-to-seven criteria, an early treatment switch to TKIs should be considered to improve their prognosis.

## 1. Introduction

Hepatocellular carcinoma (HCC) is the fifth most common cancer and the second most frequent cause of cancer-related deaths and has been estimated to be responsible for approximately 810,000 deaths per year worldwide [1]. For patients with preserved liver function, treatment procedures such as surgical resection, radiofrequency ablation, transarterial chemoembolization (TACE), and molecular targeted therapy are recommended according to their tumor stage [2]. Conventional TACE has been recommended as the first-line treatment for patients with intermediate-stage or the Barcelona Clinic Liver Cancer (BCLC) stage B HCC and preserved liver function [2]. In patients who became refractory to repeated TACE, a treatment switch to multi-targeted tyrosine kinase inhibitors (TKIs) such as sorafenib or lenvatinib has been considered [3]. However, repeated TACE has been reported to worsen liver function [4]. Deterioration of liver function from Child-Pugh grade A (CP-A) to B leads to missing the timing of treatment switch to TKIs because the efficacy and safety of TKIs are not guaranteed in patients with CP grade B (CP-B) [3,5,6,7]. Furthermore, tenacious continued TACE procedures could worsen the prognosis after switching to TKIs [4]. In fact, liver dysfunction is the main cause of death in patients with HCC [8]. Therefore, novel markers that could predict CP grade deterioration from A to B after TACE are of great importance to improve the prognosis of patients with intermediate-stage HCC.

*Wisteria floribunda* agglutinin-positive Mac-2-binding protein glycosylation isomer (M2BPGi) was recently identified as a non-invasive serum glycobiomarker for predicting the stage of liver fibrosis [9]. M2BPGi has been shown to be a reliable liver fibrosis marker in patients with chronic viral hepatitis and non-alcoholic fatty liver disease [9,10,11]. Moreover, we have previously reported that serum M2BPGi levels were highly correlated with liver function and nutritional status in patients with HCC [12]. Therefore, we hypothesized that M2BPGi could be a predictor of CP grade deterioration in patients who underwent TACE for HCC.

The aim of this study is to investigate the factors, including M2BPGi, which are associated with CP grade deterioration after TACE for HCC, and to identify the patient group for which a treatment switch from TACE to TKIs should be considered.

## 2. Results

### 2.1. Baseline Clinical Characteristics of Patients

The baseline clinical characteristics of the enrolled patients (n = 125) are shown in Table 1. The study included 96 males and 29 females (mean age, 74.3 years; age range, 41–88 years). In regard to the cause of background liver disease, there were 12 patients with hepatitis B virus (HBV), 64 with hepatitis C virus (HCV), and 49 with non-B non-C hepatitis. Nucleot(s)ide analogues were administered to all patients with HBV. Among the patients with HCV, 17.2% (11/64) had achieved sustained viral response by interferon-based therapy or direct-acting agents (DAAs). There were 69 patients with a CP score of 5 (CP-5), and 56 patients with a CP score of 6 (CP-6). Thirty patients underwent TACE as an initial treatment, while 95 patients had a treatment history of HCC. The mean follow-up period and number of TACE procedures were 15.8 months and 1.95, respectively.

### 2.2. Child-Pugh Grade Deterioration after TACE

**Figure 1:**
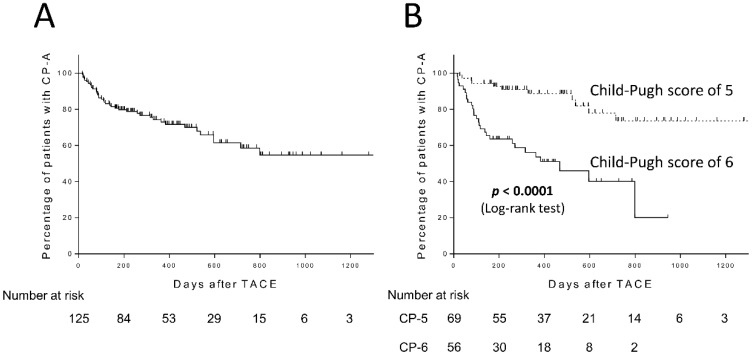
Child-Pugh grade deterioration from A to B after transarterial chemoembolization (TACE) in patients with Child-Pugh grade A (CP-A) (**A**) and patients with Child-Pugh score of 5 or 6 (**B**).

CP grade deterioration from A to B was observed in 31.2% (39/125) of the patients during the follow-up period. The cumulative CP grade deterioration rates after TACE were 20.3%, 27.1%, and 41.4% at six months, one year, and two years, respectively (Figure 1A). In regard to the number of TACE procedures, although the mean number of TACE procedures in patients with CP grade deterioration was higher than those without (2.28 vs. 1.80), they were all found to be non-significant (*p* = 0.096). We compared the cumulative CP grade deterioration rates in patients with CP-5 or CP-6. As expected, the cumulative CP grade deterioration rates after TACE in patients with CP-6 (n = 56) were 36.5% and 46.4% at six months and one year, respectively, which were significantly higher than those in patients with CP-5 (Log-rank test, *p* < 0.0001, Figure 1B).

### 2.3. Factors Associated with Child-Pugh Grade Deterioration after TACE

We next investigated the factors associated with CP grade deterioration from A to B after TACE by univariate and multivariate analyses. The Cox proportional hazards model showed that gender (female, hazard ratio (HR) = 2.97; *p* = 0.0013), albumin (ALB) ≤ 3.5 g/dL (HR = 3.48; *p* = 0.0002), total bilirubin (T-Bil) > 1.0 mg/dL (HR = 2.76; *p* = 0.0003), prothrombin time (PT) ≤ 80% (HR = 2.07; *p* = 0.0281), FIB-4 index > 4.00 (HR = 2.48; *p* = 0.0074), M2BPGi > 2.00 cut-off index (COI) (HR = 10.86; *p* < 0.0001), and beyond the up-to-seven criteria (HR = 2.35; *p* = 0.0098) were associated with CP grade deterioration after TACE (Table 2). Multivariate analysis revealed that the significant factors that were independently associated with CP grade deterioration were M2BPGi > 2.00 COI (HR = 12.41; *p* < 0.0001) and beyond the up-to-seven criteria (HR = 1.96; *p* = 0.0479) (Table 3).

### 2.4. Predictive Performance of M2BPGi for Child-Pugh Grade Deterioration after TACE

**Figure 2:**
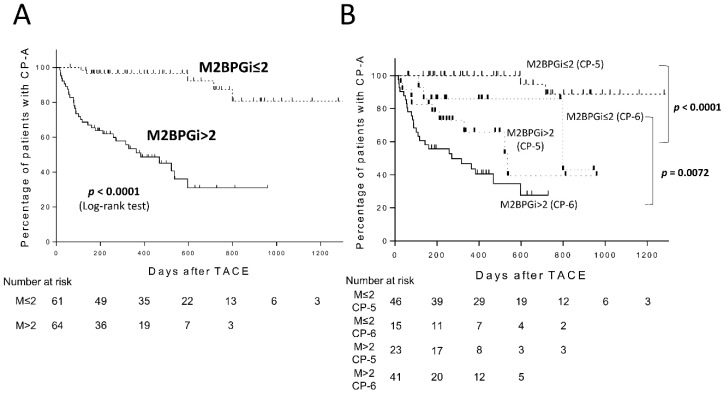
Child-Pugh grade deterioration from A to B after transarterial chemoembolization (TACE) in patients with Child-Pugh grade A (CP-A) according to Mac-2 binding protein glycosylation isomer (M2BPGi) levels (**A**) and according to M2BPGi levels and Child-Pugh score of 5 (CP-5) or 6 (CP-6) (**B**).

We performed Kaplan-Meier analysis to examine whether serum M2BPGi levels could stratify the cumulative CP grade deterioration rates after TACE. The cumulative CP grade deterioration rates of patients with M2BPGi > 2.00 COI (M2BPGi high group, n = 64) were 36.2% and 48.9% at six months and one year, respectively. Patients with high M2BPGi had a significantly faster rate of CP grade deterioration compared with those with M2BPGi ≤ 2.00 COI (M2BPGi low group, n = 61) (Log-rank test, *p* < 0.0001, Figure 2A). We further compared the cumulative CP grade deterioration rates in patients with CP-5 or CP-6 between the M2BPGi high and M2BPGi low groups. Interestingly, the cumulative CP grade deterioration rate was also significantly higher in the M2BPGi high group, regardless of the CP score (M2BPGi high and CP-5 (n = 23) vs. M2BPGi low and CP-5 (n = 46), *p* < 0.0001; M2BPGi high and CP-6 (n = 41) vs. M2BPGi low and CP-6 (n = 15), *p* = 0.0072; Figure 2B).

### 2.5. Predictive Performance of M2BPGi and Up-To-Seven Criteria Combination for Child-Pugh Grade Deterioration after TACE

**Figure 3:**
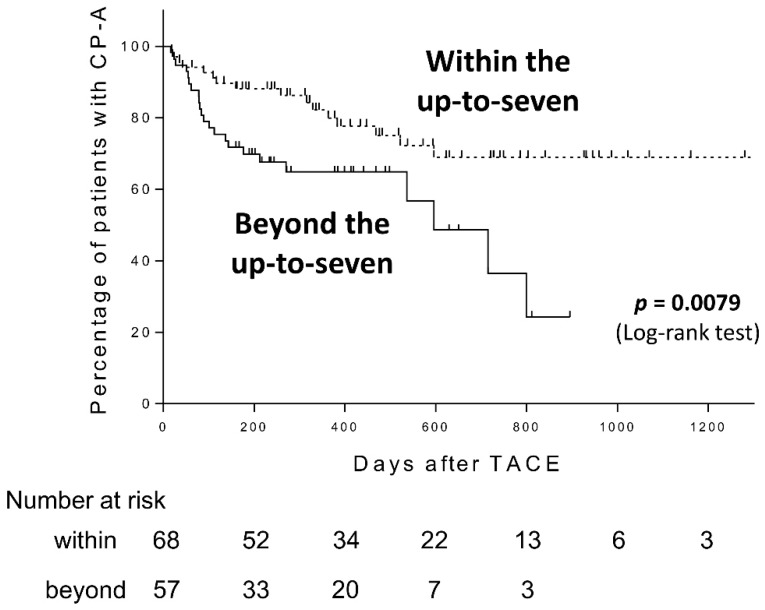
Child-Pugh grade deterioration from A to B after transarterial chemoembolization (TACE) in patients with Child-Pugh grade A (CP-A) within or beyond the up-to-seven criteria.

We also found that patients with HCC beyond the up-to-seven criteria (n = 57) had a significantly faster rate of CP grade deterioration compared with those with HCC within the up-to-seven criteria (n = 68) in our cohort (Log-rank test, *p* = 0.0079, Figure 3).

**Figure 4:**
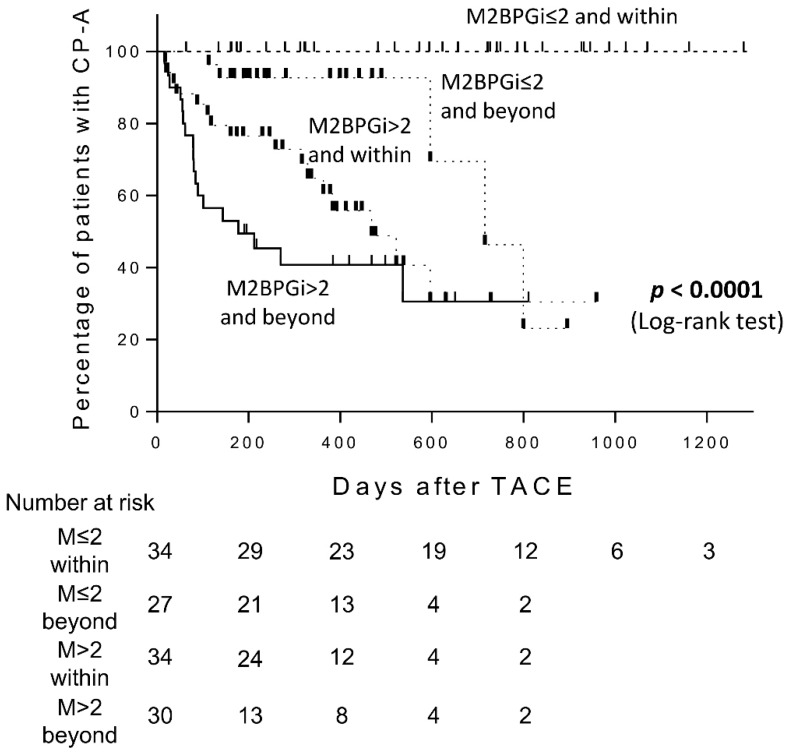
Child-Pugh grade deterioration from A to B after transarterial chemoembolization (TACE) in patients with Child-Pugh grade A (CP-A) according to Mac-2 binding protein glycosylation isomer (M2BPGi) levels and within or beyond the up-to-seven criteria.

We further investigated the predictive performance of an M2BPGi and up-to-seven criteria combination for CP grade deterioration after TACE. M2BPGi in combination with up-to-seven criteria clearly stratified the risk of CP grade deterioration after TACE (Log-rank test, *p* < 0.0001) (Figure 4). Patients with low M2BPGi and within the up-to-seven criteria (n = 34) did not experience CP grade deterioration during the follow-up period, while the cumulative CP grade deterioration rates for those with high M2BPGi and beyond the up-to-seven criteria (n = 30) were 50.6% and 59.2% at six months and one year, respectively, which were higher than for any other groups. Furthermore, although there were 10 patients with CP-5 among patients with high M2BPGi and beyond the up-to-seven criteria, the cumulative CP grade deterioration rates for these patients were higher than patients with CP-6 (36.5% and 46.4% at six months and one year, shown in Figure 1B).

Based on these findings, we suggest that the combination of M2BPGi and up-to-seven criteria could be a promising surrogate marker for early CP grade deterioration from A to B after TACE for patients with HCC.

## 3. Discussion

For patients with intermediate-stage or BCLC stage B HCC, conventional TACE has been considered as effective and recommended as the first-line treatment [2]. In patients who became refractory to repeated conventional TACE procedures, continuation of TACE using other anticancer agents or drug-eluting beads, or switching to multi-targeted TKIs such as sorafenib or lenvatinib has been considered [3]. However, tenacious continued TACE procedures have been reported to worsen liver function and prognosis after switching to TKIs [4]. Furthermore, the overall survival (OS) of patients whose treatment was switched to TKIs due to TACE-refractory status was reported to be significantly better than those who continued to undergo TACE after becoming TACE-refractory. Ogasawara et al. reported that the median time for disease progression and OS in the conversion group (switched to sorafenib after becoming TACE-refractory) were 22.3 and 25.4 months, respectively, and 7.7 and 11.5 months, respectively, in the continued TACE group (*p* = 0.001 and *p* = 0.003, respectively) [13]. Arizumi et al. also reported that the median OS was 24.7 months in the conversion group and 13.6 months in the continued TACE group (*p* = 0.002) [14]. However, in cases with deteriorated liver function after repeated TACE, TKIs were not recommended because the efficacy and safety of TKIs for patients with CP-B was not guaranteed by clinical trials [5,6,7]. Therefore, for patients who are expected to have a deteriorating liver function after TACE, an early switch to TKIs should be considered as a means of improving their prognosis. From this point of view, the detection of surrogate markers for predicting CP grade deterioration from A to B after TACE for HCC is of great importance.

In the present study, we demonstrated for the first time that the combination of serum M2BPGi level and up-to-seven criteria were useful in predicting early CP grade deterioration after TACE. Multivariate analysis revealed that the HRs of CP grade deterioration in elevated M2BPGi levels (>2.00 COI) and beyond the up-to-seven criteria were 12.41 and 1.96, respectively. The cumulative CP grade deterioration rates of patients with M2BPGi > 2.00 COI were 36.2% and 48.9% at six months and one year, respectively, which were significantly higher than those with M2BPGi ≤ 2.00 COI (*p* < 0.0001). Importantly, the cumulative CP grade deterioration rate was significantly higher in patients with M2BPGi > 2.00 COI, regardless of the CP score. Furthermore, the combination of M2BPGi and up-to-seven criteria showed higher predictive accuracy for CP grade deterioration. The cumulative CP grade deterioration rates of patients with M2BPGi > 2.00 COI and beyond the up-to-seven criteria extended to 50.6% and 59.2% at six months and one year, respectively, which were significantly higher than for any other groups. Although there were 10 patients with CP-5 among patients with M2BPGi > 2.00 COI and beyond the up-to-seven criteria, the cumulative CP grade deterioration rates of these patients were higher than for patients with CP-6. Taken together, the combination of M2BPGi and up-to-seven criteria could be a useful surrogate marker for predicting early CP grade deterioration from A to B after TACE for HCC regardless of the CP score, which may play a role in deciding on an appropriate time to switch to TKIs.

M2BPGi was identified as a novel glycoprotein-based biomarker for staging liver fibrosis in glycoproteomic biomarker screening studies [15,16]. M2BPGi has been reported to have higher efficacy in predicting liver fibrosis than other fibrosis markers such as FIB-4 index and aspartate aminotransferase to platelet ratio index in patients with HCV infection [17]. M2BPGi is also a potential marker for predicting hepatocarcinogenesis in patients with hepatitis B treated with nucleot(s)ide analogues, and in patients with hepatitis C treated with interferon-based therapy or DAAs [18,19]. Additionally, we have previously reported that serum M2BPGi levels correlated with liver function and nutritional status markers such as serum ALB concentration, the branched-chain amino acid to tyrosine ratio, controlled the nutritional status score, and the CP score in patients with chronic liver disease [12]. Moreover, the association between M2BPGi and nutritional status was especially high in patients with HCC. In the present study, serum M2BPGi levels predicted CP grade deterioration after TACE, which verified the utility of M2BPGi in the assessment of liver function in patients with HCC.

Yasui et al. previously documented the utility of the up-to-seven criteria in predicting CP grade deterioration after TACE [20]. In agreement with their report, patients beyond the up-to-seven criteria showed a significantly higher cumulative CP grade deterioration rate than those within the up-to-seven criteria. The up-to-seven criteria were initially advocated by Mazzaferro et al. for patients who underwent liver transplantation [21]. They reported that patients with HCC within the up-to-seven criteria achieved a five-year overall survival of 71%, which was similar to those who fulfilled the Milan criteria (single nodule ≤5 cm, or three nodules ≤3 cm). Because intermediate-stage (BCLC stage B) HCC comprises of a heterogeneous population of patients with a wide range of tumor burdens, sub-classification of BCLC stage B HCC using the up-to-seven criteria has recently been advocated. Kimura et al. reported that OS and disease-free survival rates were significantly higher for patients within the up-to-seven criteria compared with those beyond (*p* = 0.034 and *p* = 0.001, respectively) [22]. In patients with HCC beyond the up-to-seven criteria, the dose of anticancer agent for TACE and the administration range in the liver tends to increase, which could lead to liver function deterioration.

In the present study, female patients showed a higher risk of hepatic deterioration, although there was no significance (*p* = 0.0515). There was an imbalance in the number of female and male (29 and 96, respectively), and the rates of CP score of 6 were 55.2% (16/29) in female patients and 41.2% (40/96) in male patients, which could lead to the trend of CP grade deterioration in female patients.

In order to improve the patient’s prognosis and quality of life, accurate assessment of HCC stage, selection of an optimal treatment strategy (consideration of tumor factor), and prevention of liver function deterioration (consideration of liver function factor) are essential. Although the up-to-seven criteria are considered useful as a tumor factor, utilizing M2BPGi as a liver function factor could contribute to more effective treatment strategy development and improved prognosis. In patients with intermediate-stage HCC, elevated serum M2BPGi levels, and beyond the up-to-seven criteria, an early switch from TACE to TKIs should be considered to improve their prognosis. However, the risk of liver function deterioration after treatment with TKIs has also been reported; therefore, early and aggressive nutritional support is necessary for patients with high risk of liver function deterioration along with treatment for HCC [23]. Moreover, further studies investigating the risk of liver function deterioration after treatment with TKIs are warranted.

Overall, our findings have potentially important clinical implications for the utility of M2BPGi and the up-to-seven criteria as predictors of CP grade deterioration after TACE. However, our study has several limitations. First, our study was a single-center and retrospective design. Second, due to the limited sample size and relatively short observation period, we did not have a sufficient number of patients showing CP grade deterioration for more robust analysis. Third, the patients enrolled in this study were relatively old and mostly with HCV-related hepatitis. Thus, caution should be exercised in interpreting our results, and further studies are required to validate our observations.

## 4. Materials and Methods

### 4.1. Study Design

In total, 125 patients with intermediate-stage (BCLC stage B) HCC and CP-A (CP score of 5 or 6) who underwent TACE at Kyoto University Hospital (Kyoto, Japan) between 2015 to 2018, and had been measured with serum M2BPGi levels just before TACE, were enrolled in the study. HCC diagnosis and treatment was based on the criteria of practice guideline [2], and all cases were presented and discussed in a multi-disciplinary board comprising surgeons, gastroenterologists, and radiologists before treatment. We collected clinical data for these patients, including liver function-related clinical markers (aspartate aminotransferase, alanine aminotransferase, PLT, ALB, T-Bil, PT, and CP score/grade), tumor markers (α–fetoprotein and des-γ-carboxy prothrombin), and liver fibrosis markers (FIB-4 index and M2BPGi). All data were measured simultaneously. In the present study, patients with chronic hepatitis, pre-cirrhosis, and liver cirrhosis were included. Although CP classification was originally used for patients with cirrhosis, we used CP grade as an indicator of liver function for all patients. FIB-4 index was calculated using the Sterling formula: age (years) × AST (IU/L)/(PLT counts (×10^9^/L) × √ALT (IU/L)) [24]. Serum M2BPGi levels were measured using the HISCL M2BPGi kit (Sysmex, Kobe, Japan) with a chemiluminescence enzyme immunoassay machine as previously reported [25,26]. The measured M2BPGi values were indexed according to the manufacturer’s instructions. Whether the patients were within or beyond the up-to-seven criteria were determined by contrast-enhanced computed tomography (CT) scan or magnetic resonance imaging (MRI) as previously reported [21,27]. This study protocol conformed to the ethical guidelines of the 1975 Declaration of Helsinki. The ethics committee of Kyoto University Hospital approved the study protocol (R1849).

### 4.2. Transarterial Chemoembolization Procedure

We performed TACE, neither transcatheter arterial embolization nor hepatic arterial infusion chemotherapy, for all patients enrolled in this study. TACE was performed using selective techniques. A 2-Fr microcatheter through a 4-Fr catheter inserted into the celiac artery through the femoral artery was used to identify the tumor-feeding branch. The microcatheter was inserted to the feeding branch as far as possible under digital subtraction angiography guidance. A mixture of an anticancer agent (epirubicin or miriplatin) and ethiodized oil (Lipiodol^®^, Guerbet, Tokyo, Japan) was administered through the tumor-feeding branch, followed by an embolization using gelatin sponge particles (Gelpart^®^, Nippon Kayaku Co. Ltd., Tokyo, Japan). The selection of anticancer agents and the dosage of epirubicin, miriplatin, and Lipiodol^®^ were decided after a discussion between interventional radiologists and hepatologists with at least 8 years’ experience.

### 4.3. Follow-Up Schedule and Retreatment Strategy

Contrast-enhanced CT or MRI was carried out as follow-up imaging after TACE every 3–4 months. When tumor recurrence was detected by follow-up imaging, retreatment was planned according to the BCLC stage [2]. Blood tests were performed every 1–3 months after TACE for assessing liver function and tumor markers. CP grade deterioration from A to B is defined as being confirmed by a blood test after one month or more from TACE and irreversible after that. To accurately evaluate the association between CP grade deterioration due to TACE, the follow-up was regarded as discontinued when systemic chemotherapy such as sorafenib or lenvatinib was started.

### 4.4. Statistical Analysis

The differences in categorical variables between the groups were analyzed by the unpaired Student’s *t* test. Survival curves were created using the Kaplan-Meier method and were compared by the log-rank test. The factors associated with CP grade deterioration were identified by Cox proportional hazards regression analysis. Multivariate analyses were undertaken using the Cox proportional hazards model to identify the independent prognostic factors of CP grade deterioration. A *p* value of <0.05 was considered statistically significant. Statistical analyses were performed using JMP^®^ Pro 14 for Windows (SAS Institute, Cary, NC, USA) or GraphPad Prism 7 (San Diego, CA, USA).

## 5. Conclusions

In conclusion, we demonstrated that the combination of M2BPGi and up-to-seven criteria could be a useful surrogate marker for predicting CP grade deterioration from A to B after TACE for HCC, regardless of the CP score. In patients with intermediate-stage HCC, elevated serum M2BPGi levels, and beyond the up-to-seven criteria, it is recommended that an early switch from TACE to TKIs as well as aggressive nutritional support should be considered for improving their prognosis.

## Figures and Tables

**Table 1:** Baseline patient characteristics.

Variable	n = 125
Age (years, range)	74.3 ± 8.86 (41–88)
Gender (Male vs. Female)	96/29
Child-Pugh score (score of 5 vs. score of 6)	69/56
Etiology (HBV vs. HCV vs. non-B non-C)	12/64/49
Treatment history (Naïve vs. recurrence)	30/95
Up-to-seven criteria (within vs. beyond)	68/57
Tumor number	4.47 ± 3.67
Maximum tumor size (cm)	4.86 ± 9.45
Number of TACE procedures	1.95 ± 1.23
AST (IU/L)	47.8 ± 30.5
ALT (IU/L)	35.1 ± 30.3
Platelets (×10^4^/μL)	14.8 ± 9.92
ALB (g/dL)	3.68 ± 0.43
T-Bil (mg/dL)	0.82 ± 0.31
PT (%)	90.5 ± 14.8
AFP (ng/mL)	3506 ± 14194
DCP (mAU/mL)	2900 ± 9532
FIB-4 index	5.59 ± 4.00
M2BPGi (cut-off index)	3.31 ± 3.01

Values are presented as mean ± standard deviation (range) or number. Abbreviations: HBV, hepatitis B virus; HCV, hepatitis C virus; TACE, transarterial chemoembolization; AST, aspartate aminotransferase; ALT, alanine aminotransferase; ALB, albumin; T-Bil, total bilirubin; PT, prothrombin time; AFP, α–fetoprotein; DCP, des-γ-carboxy prothrombin; FIB-4, Fibrosis-4; M2BPGi, Mac-2-binding protein glycosylation isomer.

**Table 2:** Univariate analysis of the factors associated with Child-Pugh grade deterioration from A to B among patients who underwent transarterial chemoembolization.

Variable	No. of Cases	Univariate Analysis	
		HR (95% CI)	*p* Value
Age (>75 vs. ≤75)	69/56	1.30 (0.69–2.50)	0.4130
Gender (Female vs. Male)	29/96	2.97 (1.55–5.57)	0.0013
ALB (≤3.5 g/dL vs. >3.5 g/dL)	48/77	3.48 (1.82–6.82)	0.0002
T-Bil (>1.0 mg/dL vs. ≤1.0 mg/dL)	28/97	2.76 (1.43–5.20)	0.0003
PT (≤80% vs. >80%)	36/89	2.07 (1.08–3.89)	0.0281
Platelets (≤10 × 10^4^/μL vs. >10 × 10^4^/μL)	38/87	1.73 (0.89–3.29)	0.1031
AFP (>100 ng/mL vs. ≤100 ng/mL)	52/73	1.61 (0.84–3.06)	0.1490
DCP (>100 mAU/mL vs. ≤100 mAU/mL)	77/48	1.56 (0.80–3.19)	0.1975
FIB-4 index (>4.00 vs. ≤4.00)	70/55	2.48 (1.27–5.23)	0.0074
M2BPGi (>2.00 COI vs. ≤2.00 COI)	64/61	10.86 (4.55–32.2)	<0.0001
Up-to-seven criteria (Beyond vs. Within)	57/68	2.35 (1.24–4.58)	0.0098

Abbreviations: ALB, albumin; T-Bil, total bilirubin; PT, prothrombin time; AFP, α–fetoprotein; DCP, des-γ-carboxy prothrombin; FIB-4, Fibrosis-4; M2BPGi, Mac-2-binding protein glycosylation isomer; COI, cut-off index; HR, hazard ratio; CI, confidence interval.

**Table 3:** Multivariate analysis of the factors associated with Child-Pugh grade deterioration from A to B among patients who underwent transarterial chemoembolization.

Variable	No. of Cases	Multivariate Analysis	
		HR (95% CI)	*p* Value
Gender (Female vs. Male)	29/96	2.00 (1.00–3.94)	0.0515
FIB-4 index (>4.00 vs. ≤4.00)	70/55	0.63 (0.28–1.48)	0.2794
M2BPGi (>2.00 COI vs. ≤2.00 COI)	64/61	12.41 (4.55–40.3)	<0.0001
Up-to-seven criteria (Beyond vs. Within)	57/68	1.96 (1.01–3.90)	0.0479

Factors that showed a *p* value of less than 0.05 in univariate analysis were used for further multivariate analysis using a step-down procedure. To avoid overfitting, the components of Child-Pugh grade (albumin, bilirubin, and prothrombin time) were excluded from multivariate analysis. Abbreviations: FIB-4, Fibrosis-4; M2BPGi, Mac-2-binding protein glycosylation isomer; COI, cut-off index; HR, hazard ratio; CI, confidence interval.
